# Efficacy of vector control tools against malaria-infected mosquitoes

**DOI:** 10.1038/s41598-019-43195-6

**Published:** 2019-04-30

**Authors:** Margaux Mulatier, Soromane Camara, Alphonsine Koffi, David Carrasco, Angélique Porciani, Fabrice Chandre, Nicolas Moiroux, Thierry Lefevre, Roch Dabiré, Serge Assi, Ludovic Phamien Ahoua Alou, Laurent Dormont, Cédric Pennetier, Anna Cohuet

**Affiliations:** 1 MIVEGEC, IRD, CNRS, Univ. Montpellier, Montpellier, France; 2 CEFE, Univ Paul Valéry Montpellier 3, CNRS, Univ Montpellier, EPHE, IRD, Montpellier, France; 3 Institut Pierre Richet/Institut National de Santé Publique, Bouake, Côte d’Ivoire; 4 Institut de Recherches en Sciences de la Santé, Bobo-Dioulasso, Burkina Faso

**Keywords:** Public health, Behavioural ecology

## Abstract

Within mosquito vector populations, infectious mosquitoes are the ones completing the transmission of pathogens to susceptible hosts and they are, consequently, of great epidemiological interest. Mosquito infection by malaria parasites has been shown to affect several traits of mosquito physiology and behavior, and could interplay with the efficacy of control tools. In this study, we evaluated, in pyrethroid resistant *Anopheles gambiae*, the effect of mosquito infection with the human malaria parasite *Plasmodium falciparum* on the efficacy of nets treated with either the insecticide deltamethrin or the repellent DEET, measuring (i) mosquito success to pass through the net, (ii) blood-feeding on a host and (iii) chemicals-induced mortality. Infection of mosquitoes at non-infectious stage did not affect their success to pass through the net, to blood-feed, nor chemicals-induced mortality. At infectious stage, depending on replicates, infected mosquitoes had higher mortality rates than uninfected mosquitoes, with stronger effect in presence of DEET. This data evidenced a cost of infection on mosquito survival at transmissible stages of infection, which could have significant consequences for both malaria epidemiology and vector control. This stresses the need for understanding the combined effects of insecticide resistance and infection on the efficacy on control tools.

## Introduction

Mosquito-borne diseases are considerable public health issues, mostly affecting populations in developing countries^1,2^. To reduce their incidence, controlling vector mosquitoes and limiting their contact with human hosts remains the most effective strategy^3^. One promising approach for achieving this goal would be to selectively target, within mosquito populations, individuals that are the most dangerous for humans^4^. Particularly, mosquitoes carrying transmissible forms of pathogens in their salivary glands (i.e. infectious mosquitoes), are of great epidemiological relevance. Yet, the implementation of control means that could specifically reach infectious mosquitoes is dependent upon an extensive knowledge of the effects of mosquito infection on its behavior and physiology. Infection by pathogens has been shown to affect mosquito phenotypic traits^5^. Malaria-infected *Anopheles* mosquitoes generally display increase attraction, biting and feeding rate^6–10^. Interestingly, these changes are often concomitant with the presence of transmissible stages of the pathogen and may contribute to increase the number of contacts between human hosts and infectious vectors^11^. These effects may lead to substantial epidemiological consequences, with transmission rates of mosquito borne pathogens being potentially much higher than expected^12^. On the other hand, infection by malaria parasites may induce fitness costs on mosquitoes, reducing their survival. This can be especially true when infection is associated with other biotic and abiotic stresses^13–15^. Consequently, all the behavioral and physiological changes associated with infection could directly impact the efficacy of control means.

To date, conventional control tools mostly rely on the use of insecticide-treated nets (ITNs), although their efficacy could be threatened by the increase of insecticide resistance mechanisms in mosquito populations^16^. Besides, repellents such as DEET (N, N-diéthyl-3-méthylbenzamide) offer a great potential both in cutaneous application, spatial use or net impregnation, and their use may become complementary with ITNs^17–19^. Despite epidemiological relevance, the potential effect of infection on the efficacy of control means has received little attention. The effect of *Plasmodium* infection on the efficacy of control tools against *Anopheles* vectors has demonstrated contrasted results; while some observations suggest that it does not affect repellency and knockdown effects induced by permethrin^20,21^, a recent study showed that infection reduces personal protection offered by ITNs^22^. Another study also reported that infection can partially restore susceptibility to insecticide among mosquitoes carrying resistance alleles^15^. Besides, DEET- induced repellency was not found to be altered in *Anopheles* mosquitoes infected with *Plasmodium*^20^. Infection by *Plasmodium* could then lead mosquitoes to overcome the deterrent effects of control means, and/or induce cumulating fitness costs for the vector together with insecticides or repellents. Altogether, these results highlight the need to better understand the effects of mosquito infection on the efficacy of control tools.

In the present study, we evaluated the impact of infection by *Plasmodium falciparum* on the efficacy of treated nets, the most common control tools against malaria mosquitoes. Two compounds were selected: the pyrethroid insecticide deltamethrin, which is broadly used in net impregnation due to its disengagement and lethal effects on mosquitoes associated with a low toxicity to humans^23^, and DEET, the “gold standard” of insect repellents, which acts both as a spatial and contact repellent, and offer as well insecticidal properties^24,25^. In laboratory-controlled experiments, we measured *Anopheles gambiae* success to find a hole and to pass through a net treated with either deltamethrin or DEET, subsequent blood-feeding on a host, and chemicals-induced mortality.

## Results

*An*. *gambiae* mosquitoes of 3 to 5 days old carrying pyrethroid-resistance alleles were experimentally infected with *P*. *falciparum*-containing blood collected in human participants in Bouaké, Ivory Coast. Prevalence of infection varied between 15 and 92% across 14 experimental infections, with a mean total prevalence of 69.77% ± 5.88. Dissection at six to seven days post-infective blood meal showed oocysts numbers per female ranging from 1 to 351 across experimental infections. A subset of mosquitoes exposed to inactivated *P*. *falciparum*- containing blood (and then considered as non-infectious) was also tested for the presence/absence of *P*. *falciparum*. None of these 11 tested mosquitoes were found positive for the presence of the parasite. Behavioral experiments were performed on mosquitoes at 6–8 days post blood-meal (6–8 dpbm), when infected individuals were carrying non-transmissible stages in their midgut (*i*.*e*. oocysts), and at 12–14 days post blood-meal (12–14 dpbm), when infected individuals were carrying transmissible stages of the parasite in their salivary glands (*i*.*e*. sporozoites). The pyrethroid insecticide deltamethrin and the synthetic repellent DEET were tested for their efficacy to reduce mosquito success to find a hole and to pass through a treated net, blood-feeding on a host after passing through the net, as well as chemical-induced mortality. For each behavioral replicate and chemical, four groups of mosquitoes were tested simultaneously with the following treatments: (i) unexposed to infection - control net (NI-Control), (ii) unexposed to infection - treated net (NI-Delta or NI-DEET), (iii) exposed to infection - control net (I-Control) and iv) exposed to infection - treated net (I-Delta or I-DEET). For deltamethrin, 1,053 mosquitoes were tested across 3 behavioral replicates at 6–8 dpbm and 3 at 12–14 dpbm using blood samples from 4 donors. For DEET, 1,687 mosquitoes were tested across 5 behavioral replicates at 6–8 dpbm and 8 at 12–14 dpbm using blood samples from 10 donors.

### Effect of mosquito infection on efficacy of deltamethrin-impregnated nets

**Figure 1:**
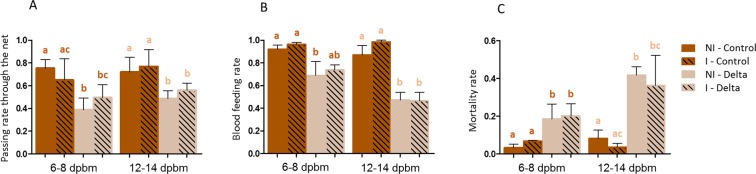
Passing rate through deltamethrin-impregnated nets (**A**), blood-feeding (**B**) and mortality associated (**C**). Blood-feeding was calculated for females that successfully passed through the impregnated net. Hatched bars show infected females, and full bars show non – infected ones. I = Infected, NI = Non-Infected. Results are presented as mean ± standard error (s.e). Different letters indicate significant differences (post hoc chi-squared tests with a Tukey correction).

Passing rate was not affected by infection at 6–8 dpbm nor at 12–14 dpbm (Table 1, Fig. 1A). Deltamethrin significantly reduced mosquito passing rate both at 6–8 dpbm (mean passing rate = 75.42% ± 7.71 in NI-Control, 38.80% ± 10.42 in NI-Delta, 65.13% ± 18.49 in I-Control and 49.58% ± 11.19 in I-Delta) and at 12–14 dpbm (72.08% ± 13.04 in NI-Control, 48.79% ± 6.90 in NI-Delta, 76.85% ± 14.93 in I-Control and 56.07% ± 6.28 in I-Delta) (Table 1; Fig. 1A).

**Table 1:** Generalized linear model output estimating the effect of mosquito infection and deltamethrin treatment on entry rate, blood-feeding and mortality associated.

Deltamethrin		6–8 dpbm			12–14 dpbm		
		*X*²	Df	P	*X*²	Df	P
Passing rate	Treatment	15.41	1	**8**.**67e**^**−5**^	6.99	1	**0**.**0082**
	Infection	0.0046	1	0.95	0.88	1	0.35
	Treatment *Infection	1.47	1	0.23	0.014	1	0.91
	Replicate	12.87	2	**0**.**0016**	9.64	2	**0**.**0081**
Blood-feeding rate	Treatment	17.77	1	**2**.**50e**^**−5**^	47.97	1	**4**.**33e**^**−12**^
	Infection	0.72	1	0.40	1.01	1	0.32
	Treatment *Infection	0.0024	1	0.97	4.52	1	**0**.**034**
	Replicate	1.18	2	0.55	4.14	2	0.13
Mortality rate	Treatment	21.10	1	**4**.**35e**^**−6**^	19.21	1	**1**.**17e**^**−5**^
	Infection	0.41	1	0.52	0.35	1	0.55
	Treatment *Infection	0.40	1	0.53	0.29	1	0.59
	Replicate	13.75	2	**0**.**0010**	0.37	2	0.83

*X*² = Chi-squared, Df = degrees of freedom, P = significance, *effect of the interaction.

Blood-feeding success was not significantly affected by mosquito infection at 6–8 dpbm, but was significantly reduced by deltamethrin treatment (mean blood-feeding = 92.24% ± 3.60 in NI-Control, 68.88% ± 12.50 in NI-Delta, 96.26% ± 1.97 in I-Control and 73.74% ± 4.40 in I-Delta). At 12–14 dpbm, a significant interaction was found between infection and treatment regarding this trait. Blood-feeding was significantly reduced by deltamethrin treatment (86.87% ± 8.44 in NI-Control, 47.27% ± 6.94 in NI-Delta, 98.55% ± 1.44 in I-Control and 46.55% ± 7.65 in I-Delta). However, paired comparisons showed only significant differences in blood-feeding due to the deltamethrin treatment and no difference between infected and uninfected females, neither upon control nets (P = 0.16) nor upon deltamethrin-treated nets (P = 0.92) (Table 1; Fig. 1B).

Mortality was not significantly affected by infection neither at 6–8 dpbm nor at 12–14 dpbm. Mortality was significantly increased in deltamethrin treatment both at 6–8 dpbm (mean mortality = 3.34% ± 1.86 in NI-Control, 18.56% ± 7.81 in NI-Delta, 6.72% ± 0.42 in I-Control and 20.19% ± 6.34 in I-Delta) and at 12–14 dpbm (8.17% ± 4.40 in NI-Control, 41.77% ± 4.47 in NI-Delta, 3.66% ± 1.90 in I-Control and 36.29% ± 15.87 in I-Delta) (Table 1; Fig. 1C).

### Effect of mosquito infection on efficacy of DEET-impregnated nets

**Figure 2:**
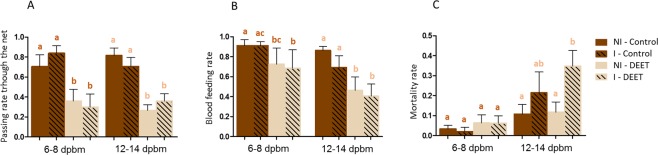
Passing rate through DEET-impregnated nets (**A**), blood-feeding (**B**) and mortality (**C**). Blood-feeding was calculated for females that successfully passed through the impregnated net. Hatched bars show infected females, and full bars show non – infected ones. I = Infected, NI = Non-Infected. Results are presented as mean ± standard error (s.e). Different letters indicate significant differences (post hoc chi-squared tests with a Tukey correction).

Infection did not significantly affect passing rate, neither at 6–8 dpbm nor at 12–14 dpbm. Passing rate was only significantly reduced in DEET treatment both at 6–8 dpbm (mean passing rate = 70.39% ± 11.82 in NI-Control, 36.04% ± 11.65 in NI-DEET, 83.78% ± 7.70 in I-Control and 29.97% ± 12.87 in I-DEET) and at 12–14 dpbm (81.52% ± 7.60 in NI-Control, 26.61% ± 5.60 in NI-DEET, 70.56% ± 8.89 in I-Control and 35.86% ± 7.33 in I-DEET) (Table 2; Fig. 2A).

**Table 2:** Generalized linear model output estimating the effect of mosquito infection and DEET treatment on entry rate, blood-feeding and mortality associated.

DEET		6–8 dpbm			12–14 dpbm		
		*X*²	Df	P	*X*²	Df	P
Passing rate	Treatment	45.48	1	**1**.**54e**^**−11**^	58.24	1	**2**.**32e**^**−14**^
	Infection	0.63	1	0.43	1.53	1	0.25
	Treatment *Infection	2.87	1	0.090	0.020	1	0.89
	Replicate	30.02	4	**4**.**85e**^**−6**^	13.95	7	0.052
Blood-feeding rate	Treatment	8.48	1	**0**.**0036**	34.54	1	**4**.**18e**^**−9**^
	Infection	0.65	1	0.42	7.48	1	**0**.**0062**
	Treatment *Infection	0.59	1	0.44	0.00	1	0.99
	Replicate	18.49	4	**0**.**00099**	26.77	7	**0**.**00037**
Mortality rate	Treatment	2.03	1	0.15	0.37	1	0.54
	Infection	0.20	1	0.65	7.22	1	**0**.**0072**
	Treatment *Infection	0.0018	1	0.97	0.60	1	0.44
	Replicate	2.69	4	0.61	31.9	7	**4**.**22e**^**−5**^

*X*² = Chi-squared; Df = degrees of freedom; P = significance; *effect of the interaction.

At 6–8 dpbm, blood-feeding was not significantly affected by infection, but was significantly reduced by DEET treatment (mean blood-feeding = 91.13% ± 5.88 in NI-Control, 72.57% ± 16.11 in NI-DEET, 91.06% ± 4.06 in I-Control and 68.57% ± 18.29 in I-DEET). At 12–14 dpbm, blood-feeding was significantly reduced both by DEET treatment and by infection. Paired comparisons showed however no statistical difference between non-infected females and infected when exposed to control nets (86.17% ± 4.11 in NI-Control and 69.17% ± 11.83 in I-Control, P = 0.063), and when exposed to DEET-treated nets (46.19% ± 13.46 in NI-DEET and 40.45% ± 12.17 in I-DEET, P = 0.098) (Table 2; Fig. 2B).

At 6–8 dpbm, mortality was not affected by infection nor by treatment (mean mortality = 3.31% ± 1.83 in NI-Control, 2.07% ± 2.07 in I-Control, 6.30% ± 4.06 in NI-DEET and 6.23% ± 3.70 in I-DEET). At 12–14 dpbm, infection showed significant effect on mortality, irrespectively of net treatment. While paired comparisons showed that infectious females displayed higher mortality when exposed to DEET that non-infected ones (11.79% ± 5.07 in NI-DEET and 34.83% ± 7.80 in I-DEET, P = 0.023), no difference was observed between infected females exposed to control net and non-infected ones (10.78% ± 4.82 in NI-Control and 21.44% ± 10.41 in I-Control, P = 0.20) (Table 2; Fig. 2C). This suggest that infection-induced mortality is higher when infectious females face DEET-impregnated nets. In order to assess whether mortality affects females before or after blood-meal, we compared mortality between non-infected and infected females among both blood-fed females and unfed ones. Infection did not significantly affect mortality among blood-fed females (*X²* = 0.046, Df = 1, P = 0.83). However, it did show significant impact among the unfed group (*X²* = 9.58, Df = 1, P = 0.0020), suggesting that mortality occurred during host seeking.

## Discussion

The results presented showed no effect of *Plasmodium* infection in mosquitoes on their ability to pass through a hole of a deltamethrin-impregnated net, to blood-feed after passing through the net, nor on their mortality. This is consistent with previous observations that did not find any impact of *Plasmodium* infection on pyrethroid efficacy^20,26^. However, it seems to contradict the recent published results that showed decreases in the efficacy of ITNs against infectious mosquitoes^22^. Some differences in the design of the assays may be responsible for these divergences. First, the chemicals used are different. They belong to two distinct classes of pyrethroids and are known to induce different effects on mosquito nervous system^27^, deltamethrin (used in the present study) being less irritant after contact than permethrin (used in the previous study)^23^. Then, we used a homozygous *kdr An*. *gambiae* strain whereas the previous study used an *An*. *arabiensis* strain partially resistant to pyrethroids. The use of specific laboratory strains under different rearing conditions could have impacted the observed results. Moreover, *An*. *arabiensis* has been shown to be more irritated by pyrethroids than *An*. *gambiae* when both strain carry insecticide resistance alleles^28^, and *kdr* mutation is also known to decrease the irritant effects of pyrethroids^28,29^. The observed effects of infection on pyrethroids efficacy in the previous study^22^ may then be impacted by the generalized insecticide resistance in vector populations^30^. The present data offer a complementary view on the effects on infection on control tools efficacy in a *kdr* homozygous population. Also, our results highlight that, although mosquitoes were carrying pyrethroid resistant alleles, deltamethrin remain effective to some extent to prevent them entering the net and to induce mortality.

The experiment testing the interactions between DEET impregnation on nets and infection status of mosquitoes showed that the presence of oocysts in mosquito midgut did not influence passing rate through the net, blood-feeding, nor mortality. In the same way, when mosquitoes harbored sporozoites, passing rate was not affected by infection under our experimental design. However, mosquitoes harboring sporozoites displayed an overall increased mortality, especially in presence of DEET. This effect of infectious parasite stages on mosquito mortality was not observed in the deltamethrin dataset, in presence and absence of the chemical. It is worth noting that a high variability was observed between replicates in the DEET dataset, so the increased mortality was not evidenced in each assay. This could be an effect of the variation in the number of mosquitoes released in the different tunnels between replicates, of blood donor, which has been shown to modulate the impact of infection on mosquito fitness^13^, and of parasite isolates.

The increased mortality observed in infectious mosquitoes in the DEET dataset, more markedly in presence of DEET, suggests that infection by *Plasmodium* could generate a fitness cost, affecting their survival. This is consistent with previous studies showing deleterious effects of infection in the *Anopheles* – *Plasmodium* system^31–33^. Although the cost of *Plasmodium* infection on mosquitoes is under debate for long time^34,35^, it appears that this cost might be expressed or amplified when associated to other stresses^13,14^ or to fitness costly mutations such as resistance alleles^36^. A stress induced by DEET could then enhance the cost of infection. Interestingly, the increased mortality was observed only in non-blood-fed females, suggesting that it occurred during host-seeking. This behavior is costly for mosquitoes^37^, and might interact synergistically with infection. Accordingly, infectious females were previously shown to display higher feeding-associated mortality, which could be a direct cost of the effort to host-seek^38^.

Our results show that, infection with *Plasmodium* does not affect ability of *kdr* resistant mosquitoes to find a hole and to pass through a net impregnated with deltamethrin. However, in some cases, infection increases mosquito mortality, especially in presence of DEET. Interestingly, when this fitness cost is expressed, it is observable only in females that harbor sporozoite stages of the parasite. This may lead to important epidemiological consequences. Yet, insecticide-resistant and infectious mosquitoes are the priority targets in the fight against mosquito-borne diseases. However, if insecticide resistant mosquitoes are more likely to die when infectious and if mortality occurs during host-seeking, transmission rates might be lower than expected. Moreover, due to their higher probability to die, those mosquitoes may be reached more easily by control tools. Altogether these observations reinforce the idea of targeting specifically infectious mosquitoes in the fight against mosquito borne diseases^39^. This could be achieved by using both lower doses of chemicals that only target infectious mosquitoes and biological control agents that act against late-life stages^40,41^. Here, DEET appears as a candidate to affect foraging behavior of epidemiologically relevant infectious females. This is strengthened by the presence a cost of DEET exposure on life-history traits in *Anopheles gambiae* females that we previously identified^42^. Nonetheless, the fact that the increased mortality we observe varied depending on replicates or studies suggests complex interactions between environment, control tools, infection and insecticide resistance. Understanding these interplays is a keystone for successfully targeting within a vector population individuals that are the more susceptible to transmit pathogens.

## Methods

### Mosquito strain and *P*. *falciparum* experimental infection of mosquitoes by Direct Membrane Feeding Assays (DMFA)

Experiments were performed on *Anopheles gambiae*. Pyrethroid resistance is widespread among malaria vector populations worldwide^30^ and the frequency of resistant alleles is particularly high in the study area^43^. Therefore, we used a KdrKis strain, fixed for the *kdr*-west allele, which confers resistance to pyrethroids and DDT. The colony was obtained by introgression into the Kisumu genome the *kdr*-west allele obtained from pyrethroid resistant mosquitoes in Kou Valley, Burkina Faso^44^. Individuals were reared at 24–28 °C and 55–80% relative humidity, with a light: dark photoperiod of 12:12 h. Larvae were reared in groups of about 300 in 1 L of distilled water and were fed TetraMin® fish food. Adults were fed a 10% honey solution for sugar intake and were maintained on guinea pigs for blood intake.

DMFAs were carried out according to procedures previously described by Ouédraogo *et al*.^45^. Screening for *P*. *falciparum* infectious human carriers were conducted in primary school groups in Bouaké, Ivory Coast. Ethical approval for the use of experimental animals and human participants was obtained from the Ministry of Health and Fight against AIDS in Ivory Coast through the National Ethic Committee N° 063/MSHP/CNER-kp. All human participants were enrolled after receipt of written informed consent from their legal guardians. All methods were performed in accordance with the relevant guidelines and regulations.

After collection of gametocyte-containing blood, plasma was removed and replaced with European naive AB serum. Batches of 3–5 days old mosquito females were allowed to feed either infectious blood or heat-treated (non-infectious) blood^46^. After one hour of exposure, unfed females were discarded and only fully fed mosquitoes were kept and maintained in the same conditions as during the rearing. Fed females were given the opportunity to oviposit. A total of 14 infectious blood samples from distinct blood donors were used.

Six to seven days after blood meal, infection rate among exposed mosquitoes was assessed in a subset of 10 females per infectious blood sample. Midguts were dissected in 0.4% mercurochrome (Sigma-Aldrich) and examined by light microscopy for the presence and number of oocysts.

### Nets impregnation and behavioral assays in tunnel tests

Pieces of polyester nets of 25 × 25 cm side were treated with either the pyrethroid insecticide deltamethrin or the synthetic repellent DEET at a dose of 25 mg/m² and 500 mg/m² respectively. Concentrations were selected to induce 25 to 75% of mean blood-feeding inhibition in order to allow for observable differences of treatment efficacy between groups of mosquitoes according to infection status. The dose used for deltamethrin also corresponds to the recommended dose for conventional net treatment^47,48^. Deltamethrin and DEET were respectively dissolved in acetone and ethanol and tested independently, using their respective solvents as controls.

Behavioral experiments were performed using tunnel tests. The equipment consists of four square glass tunnels each divided in two chambers^29,49^. In one chamber, a male guinea pig from the colony used for mosquito rearing was placed as a bait. The two tunnel compartments were separated by a cardboard frame covered with the impregnated net (25 × 25 cm). Nets were deliberately holed by nine 1 cm diameter equidistant holes to give opportunity for mosquitoes to pass from the release chamber to the second chamber containing the guinea pig. For each behavioral replicate and chemical, four groups of mosquitoes were tested simultaneously in four different tunnels with the following conditions: (i) unexposed to infection - control net (NI-Control), (ii) unexposed to infection - treated net (NI-Delta or NI-DEET), (iii) exposed to infection - control net (I-Control) and (iv) exposed to infection - treated net (I-Delta or I-DEET). The same number of females (from 10 to 120) were released in the four tunnels. Treatments were randomly attributed to each of the four tunnels at each replicate. Each behavioral replicate corresponds to an experimental infection from one given donor. For each chemical, part of the females were tested at 6–8 days post blood-meal (6–8 dpbm), when infected individuals carried oocysts in their midgut (*i*.*e*. immature, non-infectious stage of the parasite). The other part was tested at 12–14 days post blood-meal (12–14 dpbm), when infected individuals carried sporozoites in their salivary glands (*i*.*e*. mature, infectious stages of the parasite). Exposure lasted eight hours, in order to mimic one night of sleeping under a treated net, after which the position of each female mosquito in the tunnel compartments was recorded, as well as mortality at the end of the test, and blood-feeding status.

### Determination of infection status

After each behavioral assay, all females exposed to infectious blood meal and a subset of females exposed to non-infectious blood were tested for their infection status. They were conserved and stored individually in 100 µl of DNAzol® reagent (Molecular Research Center, Inc, Cincinnati, OH, USA). The entire insect was used for females killed 6–8 dpbm. Only the cephalothoraxes were used for females killed 12–14 dpbm; this allows to detect infected mosquitoes at days 6 to 8 and exclusively the presence of sporozoites stages at days 12 to 14. DNA extraction from individual mosquitoes was performed using DNAzol® according to the manufacturer’s instructions. *P*. *falciparum* detection was then carried out by qPCR^50,51^. Females were considered positive for *P*. *falciparum* when the Cq (quantification cycle) ranged from 25 to 35 and when the Tm (primer melting temperature) ranged from 75.5 to 80.

### Statistical Analysis

All statistical analyses were performed using R software^52^. Only females positive for the presence of *P*. *falciparum* were included in statistical analysis and hereafter mentioned as “infected” females. Control females exposed to non-infectious blood are hereafter called “non-infected”. DEET and deltamethrin datasets were analyzed independently, using the same statistical method. For each chemical, logistic regression by generalized linear model (glm, quasi-binomial distribution, logit link) was used to compare between infected and uninfected females: (i) the proportion of females that successfully passed through the impregnated net, (ii) the proportion of blood-fed females among those that passed the net, and (iii) the proportion of dead females. Analysis were performed separately for females at 6–8 dpbm and for females at 12–14 dpbm. Treatment (control *versus* chemical), infection status (infected *versus* non-infected), their interaction, and replicates were coded as fixed factors. Outputs of the models were obtained using type 2 ANOVA (*car* package^53^). Results are presented as mean ± standard error (SE).

## Data Availability

The datasets used and/or analyzed during the current study are available from the corresponding author upon reasonable request^54^.
